# Marine heatwaves and upwelling shape stress responses in a keystone predator

**DOI:** 10.1098/rspb.2022.2262

**Published:** 2023-01-25

**Authors:** Sarah Rühmkorff, Fabian Wolf, Jahangir Vajedsamiei, Francisco Rafael Barboza, Claas Hiebenthal, Christian Pansch

**Affiliations:** ^1^ Faculty of Mathematics and Natural Sciences-Section Biology, Christian-Albrechts-University Kiel, 24118 Kiel, Germany; ^2^ Department of Marine Ecology, GEOMAR Helmholtz Centre for Ocean Research Kiel, 24105 Kiel, Germany; ^3^ Estonian Marine Institute, University of Tartu, 12618 Tallinn, Estonia; ^4^ Environmental and Marine Biology, Åbo Akademi University, 20500 Turku/Åbo, Finland

**Keywords:** climate change, environmental fluctuations, extreme events, ecological memory, cross-stress tolerance, starfish

## Abstract

Climate change increases the frequency and intensifies the magnitude and duration of extreme events in the sea, particularly so in coastal habitats. However, the interplay of multiple extremes and the consequences for species and ecosystems remain unknown. We experimentally tested the impacts of summer heatwaves of differing intensities and durations, and a subsequent upwelling event on a temperate keystone predator, the starfish *Asterias rubens.* We recorded mussel consumption throughout the experiment and assessed activity and growth at strategically chosen time points. The upwelling event overall impaired starfish feeding and activity, likely driven by the acidification and low oxygen concentrations in the upwelled seawater. Prior exposure to a present-day heatwave (+5°C above climatology) alleviated upwelling-induced stress, indicating cross-stress tolerance. Heatwaves of present-day intensity decreased starfish feeding and growth. While the imposed heatwaves of limited duration (9 days) caused slight impacts but allowed for recovery, the prolonged (13 days) heatwave impaired overall growth. Projected future heatwaves (+8°C above climatology) caused 100% mortality of starfish. Our findings indicate a positive ecological memory imposed by successive stress events. Yet, starfish populations may still suffer extensive mortality during intensified end-of-century heatwave conditions.

## 1. Introduction

Climate change does not only lead to an overall increase in temperature [1] but also increases the frequency, duration and intensity of marine heatwaves [1,2]. Simultaneously, ocean warming intensifies the stratification of the water column. Together with eutrophication, this causes worldwide expansions of hypoxic zones [3] and facilitates the occurrences of sporadic and stressful coastal upwelling [4–6]. Heatwaves and upwelling events can commonly occur consecutively in some coastal habitats, such as the Baltic Sea [7–9]. Upwelling itself may impose multiple simultaneous changes. Such events may shoal nutrients in spring [9] and can, thus, facilitate primary production (as reviewed in Kämpf & Chapman [10]). Upwelling in late summer may provide release from heat stress, but brings water of higher salinity and will typically also be acidified (reduction in pH, increase in *p*CO_2_) and hypoxic [9]. The overall impacts of such extreme events range from single-species mortalities [11] to restructuring and losses of entire ecosystems [12,13].

Whether the succession or co-occurrence of extreme events results in additive, synergistic or antagonistic responses depend on the nature, intensity and duration, and timing of these events [14]. Recent publications have called for empirical evidence on the consequences of environmental fluctuations and the impacts of successive extreme events on marine ecosystems [13–15]. Acclimation to an extreme event may modify an individual's stress response to another succeeding pulse stress, referred to as ecological memory [15] (more precisely, ‘stress memory’ if succeeding events of similar nature are described or ‘cross-stress tolerance’ in case of succeeding events of different nature [16,17]). Thus, in contrast with a common perception of mostly negative synergistic effects imposed by multiple drivers and successive stress events [14,18], an ecological memory may mitigate the negative effects on the species to the ecosystem level.

How consecutive stressful events impact marine ecosystems remains mostly unknown (but see [14,15]). As the intensity and frequency of extremes are projected to increase [8], it is of great interest to study the main and interactive effects of such events on resident keystone and habitat-forming species. As an important and widespread benthic predator, the starfish *Asterias rubens* controls bivalve abundances in mussel beds [19] that provide habitats for numerous-associated species [20]. Disturbances of this predator–prey interaction caused by climate change and extreme events [21] can affect mussel bed formation and the functioning of associated ecosystems [22]. *A. rubens* inhabits the inter- and subtidal zones of the North Atlantic region [23–25]. Across its distribution range, *A. rubens* experiences marine heatwaves and upwelling conditions, e.g. in Chesapeake Bay, St Lawrence Bay, Long Island Sound [26,27] or in the North and Baltic Seas [28,29]. Electronic supplementary material S1 contains further details on the distribution and on temperature, salinity, acidification and oxygen tolerance of *A. rubens*.

We present an experimental study examining the consequences of the interplay between naturally occurring heatwaves and upwelling events for *A. rubens* performance, measured as feeding on mussel prey as well as their activity and body mass changes*.* We simulated four types of marine heatwaves, characterized by differences in duration and intensity (maximum intensity was at least 1°C above the threshold at which feeding ceases, see electronic supplementary material, S1), imposed on top of a climatological trajectory. After recovery from heatwaves, starfish were exposed to an upwelling event. We hypothesized that (i) the applied heatwaves would reduce the performance of *A. rubens*, with (ii) stronger impacts induced by extended or intensified heatwaves. We further (iii) hypothesized a negative impact induced by the imposed upwelling event (due to acidified and hypoxic conditions prevailing during the event) and (iv) an additive effect of both successive stress events (heatwave and upwelling).

## 2. Methods

### (a) Experimental set-up and treatments

**Figure 1.**
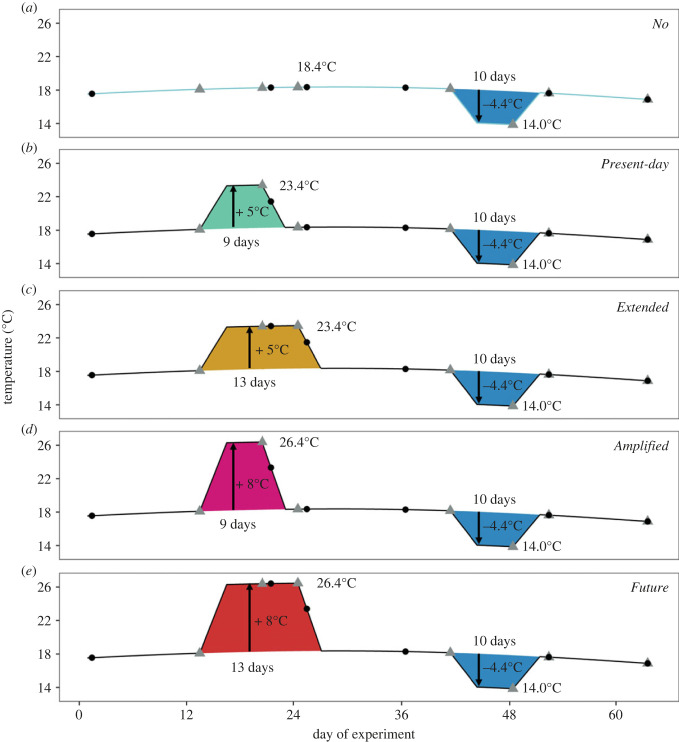
Schematic representation of the treatments experienced by individuals of *Asterias rubens* throughout the duration of the experiment. *No* heatwave: followed a smoothed natural mean seasonal temperature profile (blue line in (*a*); see methods for further information). *Present-day*: experienced a short heatwave with the intensity and duration of present-day events (9 days above the seasonal profile depicted in (*a*) with a maximum +5°C, green polygon in (*b*)). *Extended*: a heatwave of extended duration in comparison to *Present-day* (13 days above seasonal profile with a maximum +5°C, yellow polygon in (*c*)). *Amplified*: a heatwave of increased intensity in comparison to *Present-day* (9 days above seasonal profile with a maximum +8°C, pink polygon in (*d*)). *Future*: a heatwave with the combined characteristics of those described in (*c*) and (*d*) (13 days above seasonal profile with a maximum +8°C, red polygon in (*e*)). All treatments received an upwelling event (blue polygon) towards the end of the experiment, which was characterized by a drop in temperature (−4.4°C), oxygen concentration (−6.3 mg l^−1^) and pH_NBS_ (−0.5 units) as well as an increase in salinity (+2.2 units; details in electronic supplementary material, figure S1). Black dots represent measuring events of wet weight, while grey triangles represent assessments of righting responses of *A. rubens*.

We conducted our experiment using the Kiel Indoor Benthocosms [30] from 10 July until 10 September, 2018. Sixty 2 l experimental units (transparent Kautex® bottles with black lids) were evenly distributed across ten 600 l tanks, which served as water baths. In each tank, a temperature control system [30] automatically implemented five different temperature regimes (treatments were always applied in two randomly chosen mesocosms), including heatwaves and upwelling events (see further information below and in figure 1, electronic supplementary material S3: figures S1 and S5). Each of the 60 experimental units was separately supplied with fresh seawater from Kiel Fjord and received pressurized air for ventilation. Therefore, the experimental units were considered true replicates (*n* = 12).

Heatwave treatments (i.e. *No, Present-day*, *Extended*, *Amplified* and *Future* heatwaves; figure 1) were based on a heatwave characterization by Pansch *et al.* [31] and on the projected future scenarios [2]. The subsequent upwelling event, which was applied to all experimental units, mimicked an event that naturally occurred in September 2017 in Kiel Fjord (figure 1; electronic supplementary material S3: figure S5). This upwelling followed an 18 or 14 day-long recovery period from the *Present-day* or *Extended* heatwaves, respectively, and was applied for 10 days. For more details, see electronic supplementary material, S2.

Seawater temperature was measured over the entire experimental period in at least three experimental units of each tank (TTX 110 type T, Ebro, Germany). Salinity, pH and oxygen concentrations were measured along with the simulated upwelling event in all units (Multi 3630 IDS, WTW, Germany). Temperatures in the experimental units matched the targeted treatments with deviations < 0.95°C from set values and < 0.17°C among replicates (see electronic supplementary material S3: figure S1 for the entire monitoring period).

During the upwelling treatment, the temperature in the 18 l experimental units decreased from 17.8 ± 0.05°C (mean and s.d.) to 13.8 ± 0.07°C, salinity increased from 17.4 ± 0.04 to 19.6 ± 0.09, pH decreased from 7.9 ± 0.06 to 7.4 ± 0.06 (pH_NBS_ units) and oxygen dropped from 9.4 ± 0.16 mg l^−1^ to 3.1 ± 0.68 mg l^−1^ (electronic supplementary material S3: figure S5). Vaquer-Sunyer & Duarte [32] argue that 2 mg l^−1^ oxygen concentration, the threshold commonly used for defining hypoxia, is unsuitable as thresholds are highly species-specific. Indeed, the 90th percentile threshold for the median lethal oxygen concentration of marine species lies at 4.6 mg l^−1^, and for sublethal effects, even at 5.0 mg l^−1^ ([32]; see also Seibel [33]). Thus, sublethal (below 5.0 mg l^−1^) oxygen levels were experienced for 8 consecutive days in the experiment. During this time, mean temperature conditions were 14.4 ± 0.9°C (mean over all treatments and s.d.), with a mean salinity of 19.4 ± 0.3, a mean pH of 7.5 ± 0.1 and a mean oxygen concentration of 4.1 ± 1.0 mg l^−1^.

### (b) Starfish collection and measured response variables

Starfish individuals (*Asterias rubens*) were collected near Möltenort, Kiel (N54° 22′57.54″, E10°12′8.81″) on 2 July 2018. Animals were kept in a transitional tank at water temperatures measured at the collection habitat (17.6°C). After 8 days of acclimation to laboratory conditions, 12 similarly sized starfish per treatment (wet weight: 6.4 ± 1.1 g, size as arm-tip to arm-tip length: 5.5 ± 0.3 cm, mean and s.d.) were transferred to individual experimental units.

Starfish were fed *ad libitum* every third day with blue mussels (*Mytilus* spp.: 1.5–2.0 cm shell length) freshly collected from Kiel Fjord the day before feeding. After each feeding event, the shell lengths of consumed mussels (no dead mussels were observed) were measured (Dial Caliper, Wiha Division KWB Switzerland). Based on a previously described relationship between shell size and tissue dry weight for mussels in the study area [34], the dry weight of consumed mussels was estimated.

We weighed each starfish individual at the start of the experiment (day 1), during the heatwaves (day 21 for *Present-day* and days 21 and 25 for *Extended* heatwaves), in between heatwaves and the upwelling event (day 36), directly after the upwelling event (day 52) and at the end of the experiment (day 63; see also figure 1).

We measured the activity of starfish (i.e. righting response) as the time required by the individuals to turn back onto their oral side after being placed on their aboral side. Righting is essential as it maintains the individual's ability to detect and consume prey [35]. Righting measurements were performed before the beginning of the heatwave treatments (day 13), at the end of the heatwaves (day 20 and 24 for the *Present-day* and *Extended* heatwave treatment, respectively), before (day 41), during (day 48) and at the end (day 52) of the upwelling event, and at the end of the experiment (day 63; see also figure 1).

While feeding the starfish, we also checked for mortality (i.e. every third day). Starfish were considered dead if the bodies had disintegrated (electronic supplementary material S3: figure S6*c*), or if they could not move their tube feet in response to physical stimuli. One starfish in the *Amplified* heatwave treatment lost two arms (electronic supplementary material S3: figure S6*d*) and was thereafter excluded from the analysis.

### (c) Data analysis

All analyses were performed using R [36]. The impacts of the applied treatments on the performance of *A. rubens* over time and their interplay were analysed using regression approaches. Changes in the feeding rate and wet weight of *A. rubens* throughout the experiment and in response to the simulated heatwaves and upwelling events were described through generalized additive mixed models (GAMMs) fitted with the function *bam* from the R package ‘mgcv’ [37]. In addition, linear mixed models (LMM) were fitted using the function *lmer* from the ‘lme4’ package [38] to evaluate the impact of heatwave treatments over time on righting time. For feeding rate and wet weight, an additional LMM was applied using REML to test for the overall effect of the applied treatments at the end of the experiment. Electronic supplementary material S2 contains additional details regarding the statistical analyses and graphs.

## 3. Results

### (a) Survival

In our study, the survival of the starfish *Asterias rubens* differed strongly between treatments (electronic supplementary material S3: figure S7). Both *Amplified* treatments that simulated end-of-century heatwaves [39] (amplitude +8°C, maximum 26.0°C; electronic supplementary material S3: figure S1*d*,*e*) were lethal to all *A. rubens* individuals (electronic supplementary material S3: figure S7). By day 21 (i.e. 8 days of heatwave exposure), 83% and 75% of the starfish had died when the temperature exceeded 25.8°C for 3 days in the *Amplified* and the *Future* heatwave treatments, respectively (electronic supplementary material S3: figure S7). After 3 more days, all remaining individuals in both *Amplified* treatments had died (electronic supplementary material S3: figure S7). On the contrary, all *A. rubens* individuals survived exposures to *Present-day* and *Extended* heatwaves (intensity +5°C, maximum 23°C, and a duration of 9 and 13 days, respectively; electronic supplementary material S3: figures S1*b,c* and S7). The upwelling event, an abrupt change in multiple drivers (electronic supplementary material S3: figure S5), was not lethal to starfish.

### (b) Feeding rate

**Figure 2.**
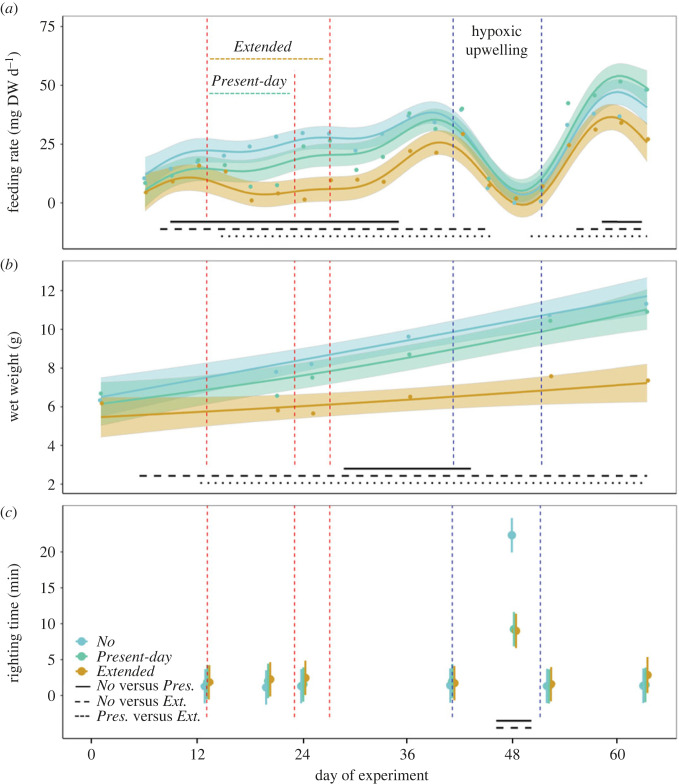
Feeding rate (milligrams of mussel dry weight per day, (*a*)), wet weight (g, (*b*)) and righting time (minutes, (*c*)) of *Asterias rubens* throughout 63 days of our experiment, under *No* (blue), *Present-day* (green) and *Extended* (yellow) heatwave treatments (see figure 1 for treatment descriptions). All treatments received an upwelling event towards the end of the experiment. The red dashed lines represent the periods of heatwaves (*Present-day* and *Extended*) and the blue dashed lines the period of upwelling. Data are represented as means (dots) of *n* = 12 experimental units. Trends in (*a*) and (*b*) were modelled using GAMM (explained deviance = 37.6% and 40.5%, respectively). Solid lines show the mean fitted trends and the shaded areas the associated 95% confidence intervals (*a*,*b*). Whiskers in (*c*) represent 95% confidence intervals. Differences between *No* and *Present-day*, between *No* and *Extended* and between *Present-day* and *Extended* are represented by solid, dashed and dotted black lines placed at the bottom of the plots, respectively (see electronic supplementary material S3: figure S8 for further details). See also electronic supplementary material S3: figures S9, S10, and S11 for related bar plots and 95% confidence intervals. Detailed statistical outcomes are presented in the electronic supplementary material S3: tables S2–S4. All starfish died after 24 days in the *Amplified* treatments and were therefore excluded from the plots.

**Figure 3.**
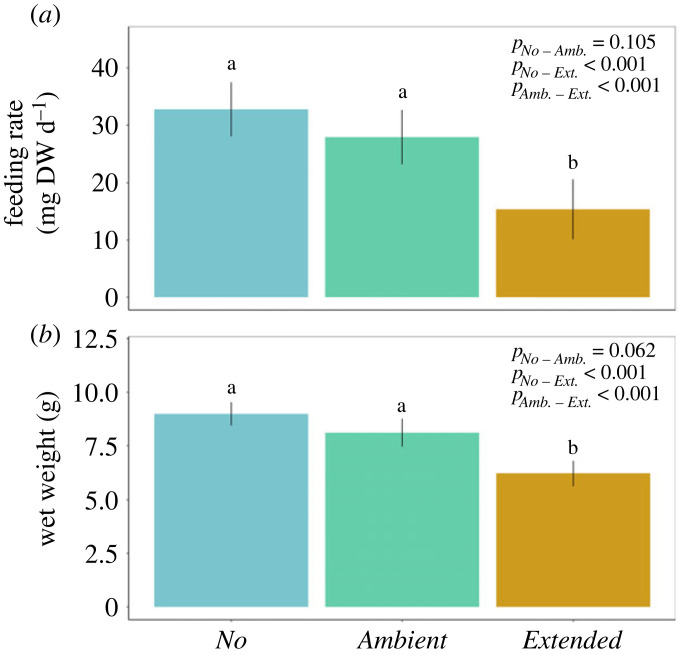
Mean feeding rate (milligrams of mussel dry weight per day, (*a*)) and wet weight (g, (*b*)) of *Asterias rubens* during 63 days of incubation, under *No* (blue), *Present-day* (green) and *Extended* (yellow) heatwave treatments (see figure 1 for treatment descriptions). All treatments received an upwelling event towards the end of the experiment. Data are presented as means and 95% confidence intervals (*n* = 12). Lower case letters represent significant differences between treatments based on LMM (see electronic supplementary material S3: tables S2 and S3). All starfish died after 24 days in the *Amplified* treatments and were therefore excluded from the plots.

Feeding rates of starfish subjected to *No* and both heatwave treatments of present-day amplitude (*Present-day* and *Extended*) closely followed the trajectories modelled by the fitted GAMM (figure 2*a*; explained deviance of 37.6%). Feeding rates in all treatments generally increased over the course of the experiment until the application of the upwelling event when feeding dropped steeply (figure 2*a*). Nevertheless, the heatwave events of the *Present-day* and *Extended* treatments significantly reduced mussel consumption by *A. rubens* (figure 2*a*) when compared to the feeding of starfish in the *No* heatwave treatment during the same period. Impacts of the *Present-day* heatwaves on *A. rubens*, however, were only transient, and the starfish could resume feeding after the heatwaves ended. Those individuals that experienced heatwaves of present-day intensity and duration consumed overall as many mussels after the event as starfish that never experienced a heatwave (figure 3*a*). By contrast, a pronounced heat-induced reduction of starfish feeding activity during the *Extended* heatwave events (figure 3*a*) caused an overall reduction of mussel consumption by 53% compared to starfish in the *No* heatwave treatment (figure 3*a*).

Simulated upwelling led to a dramatic decline in feeding rates of *A. rubens* in all treatments (figure 2*a*). Yet, starfish that had experienced heatwaves before the upwelling event on average consumed slightly (not significantly, *p* = 0.065) more soft mussel tissue, than starfish in the *No* heatwave treatment (electronic supplementary material S3: figure S12*e*). Despite the decreased feeding rate during the upwelling, this was transient, and *A. rubens* could recover from this event, and their feeding rate (enormously) increased (figure 2*a*).

### (c) Wet weight change

Wet weight of *A. rubens* linearly increased over the two-month experimental period in all three treatments as can be seen by the trajectory predicted by the GAMM, which fits the data well (figure 2*b*; explained deviance of 40.5%). No significant differences between wet weights of *A. rubens* experiencing a *Present-day* heatwave and *No* heatwave could be detected (figure 2*b*). Growth rates of starfish experiencing a *Present-day* heatwave were significantly higher than of those experiencing an *Extended* heatwave (figure 2*b*). Accordingly, the starfish's body mass in the *Present-day* heatwave treatment at the end of the summertime was not significantly impacted by the heatwave (figure 3*b*). By contrast, this trait was significantly reduced in the *Extended* heatwave treatment (figure 3*b*). Here, over the two-month experiment, wet weight of *A. rubens* was reduced by 30% (figure 3*b*) compared to the *No* heatwave treatment.

### (d) Righting time

Righting times of *A. rubens* were similar during all three treatments until the application of the upwelling event. The *Present-day* and the *Extended* heatwave had no effect on the activity (righting time) of the starfish (figure 2*c*). The upwelling, however, strongly reduced the activity (increased righting time) of *A. rubens* in all treatments. During the upwelling, however, starfish individuals that had previously experienced a heatwave were significantly more active (reduced righting time) than individuals of the *No* heatwave treatment (figure 2*c*). After the completion of the upwelling event, righting time decreased to values as low as those registered at the beginning of the experiment.

## 4. Discussion

We demonstrate that heatwaves can cause (i) either severe mortality when applying future projected intensities or (ii) temporally decrease feeding and growth of *Asterias rubens* when exposed to today's intensities, and that (iii) longer heatwaves can lead to stronger overall impacts. Furthermore, starfish (iv) strongly reduce their activity during a seemingly very stressful upwelling event. However, (v) the negative impact imposed by the upwelling event was alleviated in individuals previously exposed to heatwaves of today's intensity.

### (a) Intensity- and duration-specific effects of marine heatwaves on starfish

This experimental study showed that the performance of *A. rubens* was negatively affected by simulated marine heatwaves, and the effect strongly depended on their intensity and duration. The temperatures applied in our *Amplified* heatwave treatments (26°C) apparently exceeded the upper thermal tolerance limit of *A. rubens.* At such critical temperatures, the starfish likely suffered from a combination of extremely high cellular demands for oxygen and ATP as well as the constraints to supply those [40], potentially leading to diminished oxygen concentrations in the coelomic fluid and tissues, acute stress, tissue damage and mortality [41–44]. Extreme temperatures with peaks of 25°C were recorded in the Kiel Fjord's shallow waters in the summer of 2018 [45], just when the present experiment was being conducted. Such extreme temperatures were not only measured in the Baltic Sea, but also along the East coast of North America [46]. Thus, experimental temperatures only 1°C above this historical record in the Baltic Sea appear to be 100% lethal to *A. rubens*, presenting an emerging risk for this currently common and in places dominant, marine predator.

Peak temperatures of 23°C led to decreased starfish performance, which corroborates other recent findings: feeding rates of *A. rubens* peak at temperatures around 14°C and cease at < 2°C and > 22°C conditions (F. Melzner 2022, personal communication; [47]). In addition, the highest probability of this species’ occurrence in the Black Sea is modelled to be expected at maximum temperatures of 15°C [48]. Interestingly, recovery from these heatwaves was possible and compensatory feeding could alleviate the overall negative impact on growth. Recovery of marine species following heatwaves was also shown in previous studies [29,49] and therefore might represent a crucial aspect in species (and ecosystem) responses to climate change [49]. Although starfish tended to increase their feeding rate after an extended heatwave of the same peak temperature, they could not recover fully. Leung *et al.* [50] demonstrated that species might be resistant (i.e. no impact), resilient (i.e. recovery is possible) or sensitive (i.e. no recovery is possible) when exposed to stressful conditions. Overall, peaks of 23°C (*Present-day* and *Extended* heatwave treatments) represented conditions recorded on 18 different days in surface waters of Kiel Fjord between 1997 and 2018 [51]. This indicates that starfish are resilient to heatwaves of today's intensity but become sensitive if the stress persists longer or is of increased intensity.

Our results show that growth rates and the final size of starfish experiencing a *Present-day* heatwave were significantly increased compared to starfish experiencing an *Extended* heatwave. Therefore, a projected elongation of heatwaves by 0.5 days per decade until 2100 [2] (i.e. from 9 to 13 days) will likely negatively affect the growth of *A. rubens*, at least in the absence of thermal adaptation (see e.g. intertidal snails [50]) or the presence of temporal–spatial refugia (see e.g. *Pisaster ochraceus* [52]).

### (b) Late summer upwelling events transiently decrease starfish performance

The experimental imposition of upwelling conditions (i.e. lower temperature, higher salinity, acidification and low oxygen concentrations) reduced the performance of *A. rubens.* Ectotherms generally show a reduced metabolism at lower temperatures [53]. Still, if temperature was the dominant driver of the observed drop in feeding rate, this would correspond to a Q_10_ of 563. However, the Q_10_ of feeding is physically constrained and lies typically between 1 and 2 [54]. A previous study showed that reduced feeding rates of the starfish *P. ochraceus* during upwelling events were related to decreased temperatures with a Q_10_ of 1.8 under laboratory conditions up to 4.8 in the natural habitat [55]. As *A. rubens* optimizes its feeding at around 14°C (F. Melzner 2022, personal communication) and has the highest probability of occurrence at a maximum temperature of 15°C in the Black Sea [48], the decrease of temperature during the upwelling could not explain the considerably lowered feeding rates and would instead represent a release (temporal thermal refugia [52,56]) from the generally warm summer conditions. While the distribution of *A. rubens* is generally limited by very low salinities [57], experimental feeding rates at a salinity of 20 (i.e. during the applied upwelling) are shown to be similar to those at a salinity of 16 (i.e. conditions throughout the rest of the experiment; electronic supplementary material S3: figure S3). We, therefore, conclude that the reduced performance in the applied upwelling event should have been mainly caused by other factors than cooling or increased salinity, such as the low oxygen concentrations (3.1 mg l^−1^) and acidification (pH 7.4) in the upwelled seawater.

Experiments on the green sea urchin *Strongylocentrotus droebachiensis* have shown sublethal impacts of low oxygen at concentrations between 4.0 and 6.0 mg l^−1^ [58]. The effect of acidification on the feeding of *A. rubens* strongly depends on the acidification level applied. While feeding is not affected at an intermediate acidification level (pH = 7.8) and even shows a positive trend, feeding is negatively affected at a high acidification level (pH = 7.4; [59]), i.e. pH conditions registered during the upwelling applied in our study. Apart from single stress responses, Fontanini *et al.* [60] demonstrated that the combination of acidification (pH 7.6) and hypoxia (2.0–3.5 mg l^−1^) led to a decrease in metabolic rates of *A. rubens*. Similar negative synergistic effects of acidification and hypoxia were shown for other echinoderms [61]. Thus, activity and feeding of *A. rubens* seem to be transiently impacted during late summer upwelling events [9], most probably triggered by the acidified and hypoxic conditions in the seawater, while immediate recovery from such short-term events seems possible.

As neither acidification nor hypoxia led to mortalities during the applied upwelling, we conclude that the tested *A. rubens* population may generally tolerate moderate and transient acidification and hypoxia [59,62]. Other starfish species have also been shown to survive acidified conditions for up to four months [63] as well as short-term (3 days) hypoxia [64]. Hu *et al.* [65] discuss that under acidification, *A. rubens* allocates energy to synthesizing proteins to protect critical physiological processes. Feeding suppression under acidification (and associated changes in the carbonate system, e.g. pH, pCO_2_ and carbonate/aragonite saturation states) and hypoxia [58,66] (or high critical temperatures) potentially allows ectotherms such as *A. rubens* to allocate metabolic substrates (especially oxygen) to essential cellular processes [44]. Furthermore, potentially the lower temperature during the upwelling caused a lower metabolic rate of *A. rubens* (see e.g. Sanford [55]), which could have overall benefitted the availability of oxygen during the hypoxic conditions of the upwelling. Yet, while *A. rubens* appears temporally tolerant towards acidified and hypoxic conditions, reduced mussel consumption by the starfish, caused by upwelling (and also by preceding heatwaves) during summer months, may lead to severe reduction of starfish energy reserves, possibly decreasing the probability of long-term (across years) survival and reproduction [67].

### (c) Upwelling or spatial avoidance may provide refuge from heat stress

As was shown for other species like corals [68] and macrophytes [56], low-temperature upwelling might act as a refuge from heat stress for *A. rubens*. Therefore, despite the transient adverse effects of upwelling, these events may relieve starfish from intense heat stress (electronic supplementary material S3: figure S13*c*). During stressful upwelling events that follow (or interrupt) marine heatwaves, the habitable areas for *A. rubens*, currently in depths of 6.2–9.4 m (orange area in electronic supplementary material S3: figure S4*b*), would shift towards even shallower zones (electronic supplementary material S3: figure S4*c*). However, in the present study, heatwaves reaching the highest temperatures (up to 26°C) were lethal for *A. rubens,* whereas no mortality was observed during the applied upwelling event that entailed realistic multiple changes in abiotic drivers. As upwelling with acidified and hypoxic conditions leads to reduced activity, and as these events occur unpredictably and fast, *A. rubens* might not be able to move fast enough to escape such sublethal stress.

Maximum temperatures in surface waters (electronic supplementary material S3: figure S13*a*) tend to occur at the same time as minimum pH and oxygen concentrations in bottom waters (electronic supplementary material S3: figure S13*b*; see also [9] for details on the Kiel Fjord). During late summer, oxygen minimum zones regularly form in deeper layers of marginal seas like the Gulf of Mexico [69] or the Baltic Sea [70]. Migrating to these (cooler) waters would, thus, expose organisms to acidification and hypoxia (electronic supplementary material S3: figure S4*b*), which may reduce organismal functioning (i.e. secondary production and community maturity: [69]). In the future, more stable seawater stratification caused by extended warm periods (and heatwaves) as well as a progressing eutrophication [3,71,72] will further foster the formation of a distinct acidified and hypoxic bottom layer. Hence, the size of refuge habitats for mobile species, like *A. rubens*, will be reduced in many coastal regions (electronic supplementary material S3: figure S4*b*).

### (d) Sublethal heatwaves may induce resistance to upcoming upwelling

Contrary to expectations, starfish during the upwelling event benefited from the stress experienced previously in the form of a sublethal marine heatwave. More precisely, the activity of *A. rubens* that experienced a previous heatwave was 2.4 (*Present-day*) or 2.5 (*Extended*) times higher during the upwelling than that of naive *A. rubens* not experiencing a heatwave prior to the upwelling event. This pattern was also visible (as a strong but insignificant trend, *p* = 0.065) in recorded feeding rates of *A. rubens* (on average 2.5—*Present-day—*or 2.4—*Extended*—times higher, electronic supplementary material S3: figure S12*e*). Reductions in feeding rates during the upwelling event were dramatic and occurred across treatments, potentially masking parts of the differences between heatwave treatments. In addition, as the period during which starfish experienced acidified and hypoxic conditions was short, higher mean feeding rates did not reverse the overall pattern of smaller individuals found in the *Extended* heatwave treatment. Typically, smaller sized benthic invertebrate taxa are found in areas with regularly occurring hypoxia [73]. A higher surface-to-volume ratio results in a larger diffusive boundary layer through which more oxygen can be acquired in skin-breathing animals like *A. rubens*. Therefore, the smaller sized starfish that resulted from the *Extended* heatwave could have had an advantage during the subsequent upwelling. Furthermore, we qualitatively observed that the starfish's arms became longer and thinner during the upwelling event (electronic supplementary material S3: figure S6*a* compared to figure S6*b*). This morphological change might have affected gas exchange, a finding that requires further investigation.

Theory suggests that the impacts of upwelling as a subsequent natural stressor could be mitigated to some extent by a preceding stress event [15,17]. As starfish were of similar size in the *No* heatwave and the *Present-day* heatwave treatments, morphological (size) variation cannot explain the higher activity (and partly feeding rate) of starfish in heatwave versus no heatwave treatments (see discussion above). Starfish previously exposed to heatwaves might have required energy and therefore fed even during the upwelling event. More plausibly, acclimation to heatwaves could have caused physiological and behavioural adjustments that functionally prepared starfish for the upwelling (i.e. ecological memory or cross-stress tolerance [15,17]). In particular, cross-stress tolerance enables species, after exposure to an initial stressor, to better tolerate a subsequent stressor of a different nature [16,17].

Several studies have highlighted the role of heat shock proteins (HSPs) in cross-stress tolerance in terrestrial plants and fish species [74–76]. However, the underlying mechanisms are not yet fully understood. Potentially, genetic and molecular modifications are involved. Activation and upregulation of heat shock factors (e.g. HFS1) and hypoxia-inducible factor HIF1a lead to an increased expression of HSPs [77–79]. These interactions between HIF1a and HSPs could explain the cross-stress tolerance between heat and hypoxia [77–79]. HSPs have also been shown to play an essential role in the response of marine species to acidification (as reviewed by Yusof *et al.* [80]). Therefore, the expression of transcription factors activating HSP genes and, thus, upregulation of HSPs during heatwaves could have also been beneficial for the performance of *A. rubens* in response to the applied upwelling event.

## 5. Conclusion

Our work demonstrates that short-term—but extreme—pulse events can significantly impact marine species. Noteworthy, the strength of the impact from heatwaves strongly depends on the amplitude and duration (i.e. overall strength) of the heatwave event. While upwelling entails multiple changes, acidification and oxygen deficiency likely represent the primary drivers reducing *A. rubens* activity. Consequently, heatwaves and upwelling will temporally reduce the *in situ* feeding pressure of this key predator, *A. rubens,* on mussel beds [22], possibly having cascading ecosystem-wide consequences in the Western Baltic Sea and potentially other temperate regions of the Northern Atlantic region [22,81,82]. The successive occurrence of stress events of different natures and the concepts of ecological memory and cross-stress tolerance are theories already intensively studied in plant ecology (e.g. [16,17]). However, we are only starting to understand such phenomena in the marine realm. The present study highlights such cross-stress tolerance enabling starfish to endure and withstand consecutive stressors of differing quality (heatwaves versus upwelling) and to potentially acclimate to changing and fluctuating environments in the future. Overall, this study demonstrates the general importance of considering environmental fluctuations in experimental ecology and stresses the necessity for evaluating the concomitant effect of extreme events to generate realistic projections of how marine ecosystems may be transformed during climate change.

## Data Availability

Data collected during the experiment are available on PANGAEA: https://doi.pangaea.de/10.1594/PANGAEA.930929. The electronic supplementary material is available online [83].
